# Service-level and practitioner-level recommendations for better care of people with dementia who live alone and have no informal caregivers

**DOI:** 10.1371/journal.pone.0358131

**Published:** 2026-10-09

**Authors:** Claudio Di Lorito, Fiona Aspinal

**Affiliations:** 1 Department of Primary Care and Population Health, University College London, London, United Kingdom; 2 School of Medicine, Keele University, Keele, United Kingdom; University of New Brunswick, CANADA

## Abstract

**Context:**

A substantial proportion of people with dementia live alone without informal caregivers, yet services often assume family involvement, increasing risks of unmet need and adverse outcomes.

**Objective:**

This study aimed to generate service-level and practitioner-level recommendations to best support people with dementia who live alone and lack informal caregivers.

**Methods:**

A qualitative phenomenological co-design workshop was conducted with seven practitioners from statutory, voluntary, and private-sector roles across England. Prompts informed by earlier qualitative work were used as a template to stimulate reflection and develop recommendations for practice through inductive thematic analysis and input from a study advisory group including people with lived experience of dementia.

**Findings:**

Practitioners recommended some service-level and practitioner-level recommendations. Service-level recommendations included flexible care pathways and sufficient time to build rapport in the absence of informal support. Practitioner-level recommendations, approaching the client with personability, humanity, warmth, respect, and humility, and adopting a sensitive yet honest communication, were essential for meaningful engagement and supporting autonomy. Curiosity and creativity helped practitioners understand behavioural motivations and tailor interventions. Experience underpinned all these competencies, with supervision and shadowing seen as vital for developing relational skills.

**Limitations:**

The study sample was small, although it included a diverse range of practitioner backgrounds. No people living with dementia participated directly in the study, although people with lived experience of dementia were involved in the study advisory group.

**Implications:**

High-quality support for people with dementia who live alone requires service structures that enable good practice from practitioners. Embedding flexibility, time investment, and relational practice within dementia services is essential to enable effective, person-centred care, particularly where formal systems must compensate for the absence of informal caregivers. These prerequisites are critical conditions to ensure equitable support for people with dementia who live alone.

## Introduction

Dementia care in the community is underpinned globally by the contribution of informal caregivers, most commonly family members, friends, or neighbours [1]. Informal caregivers provide extensive practical, emotional, and social support [2] and frequently act as facilitators in navigating health and social care systems as cognitive impairment progresses [3]. Therefore, much of the evidence informing dementia care policy, commissioning, and best practice is based on the assumption that an informal caregiver can coordinate or supplement formal support [4].

However, this assumption does not hold for all people with dementia. A substantial proportion of individuals living with dementia do not have regular informal support or may have social networks that are unable or unwilling to provide even basic care [5]. Estimates suggest that around 40% of people with dementia living in the community in the UK live alone, and up to one in ten of these individuals have little or no informal support [6]. Demographic trends, including increasing numbers of older adults living alone and ageing without children, indicate that the population of people with dementia without informal caregivers will continue to grow [7].

A wide range of services contribute to supporting people with dementia who live alone and have no informal caregivers, including social care, occupational therapy, and social prescribing. These aim to offer support with personal care, home care, reablement, day services, social opportunities, home adaptations, and information and advice [8]. Yet despite the concerted efforts, recent research found that people with dementia who live alone still face great challenges in accessing, using, and adjusting support as their needs change, which can increase the risk of unmet need, lack of (adequate) care arrangements and lead to poorer health outcomes [9].

Given the distinct challenges in working with this client population, in our previous study we identified strategies that practitioners supporting people with dementia who live alone and have no informal support found effective [10]. Using those strategies as a template for discussion, this study aims to co-develop with practitioners some practical recommendations to inform best practice and contribute to improving support for this growing and underserved group of people living with dementia.

## Materials and methods

### Design

This was a qualitative phenomenological study comprising a co-design workshop with practitioners with direct experience of supporting people with dementia who live alone and have no informal support. It was part of a broader research project also including:

- A scoping literature review to identify approaches to enhance engagement with service interventions among people living with dementia [11]
- A qualitative, interview‑based study to identify strategies to best support clients with dementia who live alone and have no informal support throughout the dementia care pathway [10]

The study followed the Standards for Reporting Qualitative Research (SRQR) [12] (Table 1).

**Table 1:** Standard for Reporting Qualitative Research (SRQR) (O’brien et al., 2014).

Title and abstract	Page
**Title** – Concise description of the nature and topic of the study Identifying the study as qualitative or indicating the approach (e.g., ethnography, grounded theory) or data collection methods (e.g., interview, focus group) is recommended	**1**
**Abstract** – Summary of key elements of the study using the abstract format of the intended publication; typically includes background, purpose, methods, results, and conclusions	**2**
**Introduction**	
**Problem formulation** – Description and significance of the problem/phenomenon studied; review of relevant theory and empirical work; problem statement	**4**
**Purpose or research question** – Purpose of the study and specific objectives or questions	**5**
**Methods**	
**Qualitative approach and research paradigm** – Qualitative approach (e.g., ethnography, grounded theory, case study, phenomenology, narrative research) and guiding theory if appropriate; identifying the research paradigm (e.g., postpositivist, constructivist/ interpretivist) is also recommended; rationale**	**5**
**Researcher characteristics and reflexivity** – Researchers’ characteristics that may influence the research, including personal attributes, qualifications/experience, relationship with participants, assumptions, and/or presuppositions; potential or actual interaction between researchers’ characteristics and the research questions, approach, methods, results, and/or transferability	**5**
**Context** – Setting/site and salient contextual factors; rationale	**11**
**Sampling strategy** – How and why research participants, documents, or events were selected; criteria for deciding when no further sampling was necessary (e.g., sampling saturation); rationale	**11**
**Ethical issues pertaining to human subjects** – Documentation of approval by an appropriate ethics review board and participant consent, or explanation for lack thereof; other confidentiality and data security issues	**16**
**Data collection methods** – Types of data collected; details of data collection procedures including (as appropriate) start and stop dates of data collection and analysis, iterative process, triangulation of sources/methods, and modification of procedures in response to evolving study findings; rationale	**12**
**Data collection instruments and technologies** – Description of instruments (e.g., interview guides, questionnaires) and devices (e.g., audio recorders) used for data collection; if/how the instrument(s) changed over the course of the study	**12**
**Units of study** – Number and relevant characteristics of participants, documents, or events included in the study; level of participation (could be reported in results)	**17**
**Data processing** – Methods for processing data prior to and during analysis, including transcription, data entry, data management and security, verification of data integrity, data coding, and anonymization/de-identification of excerpts	**12**
**Data analysis** – Process by which inferences, themes, etc., were identified and developed, including the researchers involved in data analysis; usually references a specific paradigm or approach; rationale**	**16**
**Techniques to enhance trustworthiness** – Techniques to enhance trustworthiness and credibility of data analysis (e.g., member checking, audit trail, triangulation); rationale	**16**
**Results/findings**	
**Synthesis and interpretation** – Main findings (e.g., interpretations, inferences, and themes); might include development of a theory or model, or integration with prior research or theory	**17-23**
**Links to empirical data** – Evidence (e.g., quotes, field notes, text excerpts, photographs) to substantiate analytic findings	**17-23**
**Discussion**	
**Integration with prior work, implications, transferability, and contribution(s) to the field –** Short summary of main findings; explanation of how findings and conclusions connect to, support, elaborate on, or challenge conclusions of earlier scholarship; discussion of scope of application/generalizability; identification of unique contribution(s) to scholarship in a discipline or field	**24-27**
**Limitations** – Trustworthiness and limitations of findings	**26**
**Other items on the SPQR are reported in the paper**	

### Sample

The sample comprised practitioners in England who met the following criteria:

- Employed in any sector, including statutory, voluntary, or private organisations
- Working in any practitioner role (e.g., social prescribers, social care practitioners, admiral nurses, voluntary‑sector staff)
- Having direct experience of supporting people with dementia who live alone and lack informal caregivers
- Aged 18 or over, resident in England, and willing and able to provide informed consent and participate in a group workshop.

### Recruitment

Practitioners who took part in the previous qualitative interview study [10] were re-contacted and invited to take part in the workshop. These had been initially identified through the research team’s networks across England, including contacts within social care services, social prescribing teams, primary care networks, local authorities, and organisations in the voluntary and private sectors. Recruitment for this study started on 2.1.2025 and ended on 2.12.2025.

While convenience sampling was adopted to recruit study participants, we monitored socio-demographic and practitioner characteristics of participants to promote diversity in the sample. We initially anticipated recruiting up to eight practitioners to achieve sufficiently rich, varied, and multi‑layered perspectives, as per current guidance [13], but the final decision on sample size was based on pragmatic aspects, including date/time set for the session and availability of practitioners to take part on the agreed/set date/time.

### Data collection

Written informed consent was obtained from all participants prior to the start of the workshop, using electronic consent forms. Participants also completed a demographic questionnaire in advance of the session. The workshop was conducted remotely via Microsoft Teams to enable participation from across England and to accommodate practitioners’ schedules. It took place on 9 December 2025, 10:30–12:00. The session was co-chaired by the two authors, who shared roles and responsibilities (e.g., asking prompts, facilitating the discussion, managing the chat function, taking notes). The session was video‑recorded using Microsoft Teams. A transcript was automatically generated.

### Materials

The workshop was guided by initial prompts informed by the strategies identified in the preceding qualitative interview study [10] (Table 2). The strategies were presented to the group and used as a template to stimulate reflection and develop recommendations for practice.

**Table 2:** Challenges and potential strategies for best practice with people living with dementia who live alone and have no caregivers used as template for discussion in the group workshop. Adapted from Di Lorito and Aspinal [10].

Challenge	Strategy
Identifying relevant clients	• Contact people with at-risk demographics (e.g., aged 55+ and living alone) for regular ‘welfare calls’ • Embed dementia screening in chronic conditions monitoring and management reviews • Appreciate importance of informal disclosures to non-clinical staff (e.g., receptionists) • Maximise community outreach through, for example, weekly clinics • Implement rapid response (e.g., a hotline number)
Overcoming stigma about dementia/support and practitioner role	• Substitute sensitive terms (e.g., dementia, caregiver, memory test, with more acceptable ones (e.g., ‘memory’, ‘someone helping out’, ‘general check-up’), if needed • Phrase questions that elicit a sense of agency (e.g., ‘if we could do anything, what would you want?’) • Use practical examples to explain your role
Building the ‘truth’	• ‘Test’ the consistency of answer by asking the same question at different times • Rely on observational methods—such as noting hygiene, odours, and food preparation vs asking remotely though phone
Sharing information between services	• Make extensive notetaking to pass to practitioner taking over • Make joint visits with person who knows the client before taking over
Overcoming barriers to engagement	• Shift from an emphasis on independence toward one of interdependence • Use traditional methods of communication over technology (e.g., post letters, take notes, knock on door) • Take time to listen to and explore with your client, as barriers to motivation may be hidden • Show decision-making power is shared • Build trust with clients through consistent (i.e., same person over time) support and personalised interactions (e.g., asking about pets on every visit) • Being straightforward about the risks of hospitalisation, if support is refused
Moving from risk-aversion to risk-management	• Challenge conventional ideas on age and sexual intimacy and adopt non-ageist, inclusive, person-centred approaches • Make environment assessment in person • Assess functional ability by asking client to undertake some activities in front of you (e.g., make me some tea) • Adopt a more dynamic approach that balances autonomy with safety (i.e., risk management VS risk elimination) • Make technology support understandable and accessible (i.e., installation, explanation, follow up)
Strategies for continuity of care	• Interdisciplinary communication between referrer and referee, i.e., talk to each other regularly • Maintain involvement beyond discharge/referral, i.e., hold regular follow ups • Reassure/inform clients being discharged that they can come back to you • Make extensive notetaking to pass to practitioner taking over • Make joint visits with person who knows the client before taking over • Prioritise consistency, adaptability, and long-term support over short-term rigid service boundaries

### Data analysis

Transcripts produced by Microsoft Teams and the chat from the workshop were checked for accuracy, corrected where necessary, anonymised by the first author, and assigned non‑identifiable participant codes such as P01 and P02. Reflexive Thematic Analysis [14] with an inductive approach guided the analytic process. Initial themes (i.e., recommendations for best practice) were generated as they emerged from the workshop data. The initial themes were revised, merged, condensed, or removed as needed through collaborative work of the two authors. Preliminary sub-themes within the themes were also generated. The set of themes/sub-themes was then reviewed by a study advisory group. The group comprised two Patient and Public Involvement (PPI) collaborators with dementia living alone and with no informal support from underserved communities: Black British, and Lesbian, Gay, Bisexual, Transgender, Questioning/Queer and other sexually minoritised groups (LGBTQ+). It also included two researchers who conducted previous work in this area, one social worker working and a commissioner of Adult Social Care services working respectively in two Local Authorities in England. The themes/sub-themes were presented to the advisory group, who offered their views around relevance and completeness, and worked collaboratively to refine and co-develop a final set of practical recommendations to inform best practice.

### Ethics

The study obtained sponsorship from UCL, and HRA (Health Research Authority)/IRAS (Integrated Research Application System) ethical approval from the Social Care Research Ethics Committee (Ref: 24/IEC08/0033). As per ethical requirements, written consent was obtained from the participants prior to the workshop.

## Results

Seven participants took part in the workshop, of whom four were female and three were male. The sample was diverse in terms of professions, including one social prescriber working in general practice and one working in hospital, one clinical nurse specialist in dementia and delirium working in hospital, one admiral nurse working in the community, one academic social worker working for a local council, one director of dementia home care working in the private sector, and one occupational therapist working for a local council. As per our inclusion criteria, all participants had direct experience of supporting people with dementia who live alone and have no informal caregivers. All participants worked in the greater London region, except for one, who worked in Yorkshire.

All the strategies [10] initially presented to the participants were found to be relevant. However, the participants felt that, in order to be applicable and deployable, the strategies need some pre-requisites, both at the service and practitioner level. These pre-requisites were the core recommendations for practice that emerged from the workshop and are presented below.

### Recommendations for services

Two main service-level recommendations were identified: flexibility and time investment.

#### Flexibility.

Flexibility in dementia service configuration and practice is essential to respond to the distinct needs of clients with dementia who live alone and have no informal caregivers. Unfortunately, at present, services denote a lack of flexibility, with clients with dementia who live alone and unsupported being often “presented with limited options, which they either take or they are discharged” (P02, social prescriber). This does not take into account the fact that an initial resistance to accepting support (e.g., to take part in a community activity) may conceal ulterior barriers (e.g., embarrassment about incontinence) that have nothing to do with the client’s unwillingness to engage. This could be addressed by adopting a more adaptable, person-centred approach, exploring the underlying issue, and making small adjustments.

The lack of flexibility seems to underline an assumption about the typical characteristics and presentations of clients with dementia, which fails to recognise that those who live alone and are completely unsupported may not fit the rigid pathways of services. A classic example was reported by one practitioner who noted that services generally “assume that people with dementia are old” (P01, social prescriber), but one of her clients who lived independently and unsupported had young onset dementia, and the service was not well prepared/equipped to respond flexibly to this client’s unique needs (e.g., navigating work systems, seeking intimacy and the resulting implications in terms of risks).

In essence, flexibility is crucial because rigid service pathways fail to accommodate the diverse and often hidden needs of people with dementia who live alone, underscoring the importance of adaptable, person-centred approaches.

#### Time investment.

In order to appreciate the nuances of a client’s needs, another key service-level feature should be time investment. In the absence of informal caregivers, practitioners reported that rapport building is a slow process that occurs over time. One practitioner found that “it actually takes several visits” for some individuals to feel ready to engage, particularly when they are “struggling with accepting the diagnosis” (P02, social prescriber). Another practitioner reported that they “take some time to explain what those terms mean… to demystify these things” (P03, social worker) including discussing the origins and meaning of the term dementia to reduce stigma and improve understanding.

Time spent with clients in everyday home-based activities such as making tea, was found to facilitate natural conversation and break down barriers to engagement more effectively than formal interviews/assessment. Accompanying clients to structured activities in the community were also described as valuable, as explained by one practitioner: “dementia friendly or inclusive activities are great because during those visits we actually can build up some rapport, some good working relationship” (P02, social prescriber).

Because of the importance of time investment, some practitioners reported that in their services there is an increasing effort to embed time needed to build “rapport [as] an integral part of the rehab plan” (P04, Occupational therapist). This approach sees practitioners as active contributors to rehabilitation in a more holistic sense rather than as mere task-focused service providers.

To sum up, fostering meaningful engagement with people with dementia who live alone requires time investment. It is crucial to building rapport and reducing stigma, which demands repeated, informal, and relationship-focused interactions over time.

### Recommendations for practitioners

Five main practitioner-level recommendations were identified: personability and humanity, respect and humility, sensitivity and honesty, curiosity and creativity, and experience.

#### Personability and humanity.

The practitioners reported that some practitioner-level qualities are also required to provide better care to people with dementia who live alone and have no informal support. A key one is the ability, willingness and disposition to be personable and human, to treat the client with dignity and as an individual. While it may sound obvious that interacting with the client promotes dignity in care as well as good rapport and human connection, a practitioner reported that “a lot of people tell me that carers do their job mute” (P05, admiral nurse).

Being open to talk and establish a human connection is particularly relevant when working with people who are at risk of social isolation, as it was observed that the practitioner “might be the only person that the person living with dementia sees that day” (P05, admiral nurse). It is therefore important to be able to “be personable and not create too much practitioner distance” (P03, social worker), and to grasp some of the topics that might catch the interest of the client and engage them. One practitioner brought an example of connecting on a shared experience with their client: “I have a client with dementia who is Scottish, she loves me now as I picked up on that and shared my experience of how much I loved living in Scotland” (P03, social worker).

Together, these insights highlight that authentic human connection is not just a supportive skill but an essential foundation for effectively engaging and supporting people with dementia who live alone and without informal care.

#### Respect and humility.

Being able to take the necessary time to build rapport, trust and relationships with clients, practitioners were also able to understand the person’s individual capacity and social assets. This led to better acknowledgement of the client’s preferences and respect for their agency and autonomy.

Another essential quality to achieving person-centred care is for practitioners to have the humility to recognise that lived experience is what really makes someone the expert in dementia care needs. As explained by one practitioner: “I am a specialist in dementia delirium but I’m not an expert. I don’t live with dementia. This is the difference. I can tell you A to Z what you might get in terms of symptoms, but I will never be able to tell you the real deal in terms of day in, day out, what dementia feels like for me and how that stops me or helps me interact better with those around me and managing my life” (P06, clinical nurse specialist in dementia and delirium).

An example of a respectful approach to fulfilling the client’s individual wishes and needs related to communicating a diagnosis. A practitioner commented: “In principle, it is important to communicate it, but also we need to remember that it is what really counts or matters for the person themselves, because there are those who have explicitly expressed that they wish not to know” (P06, clinical nurse specialist in dementia and delirium). Another practitioner provided a second example of a respectful approach to client’s wishes around social support: “A lot of people living alone with dementia might have always been what we might call socially isolated, but they’re just people who like their own company. So just because they develop dementia and we come across them, trying to funnel them into group activities and doing stuff in groups is often not appropriate” (P03, social worker).

Thus, through taking a humble, respectful, person-centred approach, practitioners could support their clients with dementia without family, to retain agency in decisions about their care, their needs and their social lives

#### Sensitivity and honesty.

The tension potentially rising between practitioners’ views on good practice and respecting the client’s wishes requires a delicate balance between sensitivity and honesty. The example provided by practitioners were around communication, particularly around the use of sensitive terminology. One practitioner recognised that while fulfilling their wishes, “you have to be careful that you are not misleading people. So, for example, for a memory test, if you’re asking someone if they want to be referred for it, you have to call it what it is at some point in the proceedings” (P01, social prescriber), both from an ethical and a legal standpoint.

Continuing with the same scenario, a practitioner reported finding it effective to lower the stigma attached to a memory test by stressing its usefulness, and hinting that many people share the same situation: “talking to other people in your situation, they find x, y, z as a starting point” (P03, social worker).

Adopting a sensitive yet honest approach to communication also includes simplifying concepts and questions so that clients can grasp them, avoiding feelings of disempowerment that could lead to disengagement, as reminded by one practitioner: “You also have to be mindful of asking questions that people will find hard to answer as that can be anxiety provoking and people can close down...” (P07, director of dementia home care from the private sector).

In conclusion, supporting people with dementia who live alone requires a careful balance of sensitivity and honesty, ensuring clear, stigma-reducing communication that respects clients’ wishes while avoiding misinformation and preventing disengagement through accessible, non-anxiety-provoking interactions.

#### Curiosity and creativity.

Another important quality that characterises good practice is practitioners’ ability to be curious and creative. Being able to go deeper than the surface level to explore clients’ reasons for certain behaviours is crucial when working with people with dementia who live alone and have no informal caregivers, as found by one practitioner: “What I try to do is tap into what’s going on behind the behaviour. So, for example, if people with dementia go out on their own and they think they’re going to their parents when their parents are dead, we assume it’s part of the dementia. But at the same time, quite often it’s because they feel insecure at home and they’re actually consciously or subconsciously seeking somebody to provide reassurance. So, I just try and look into what’s behind it” (P03, social worker).

Adopting a creative approach when supporting these clients is another quality that the practitioners found important: “Sometimes it’s important to be a bit creative and seize the moment. For example, I rang a client for the first time to arrange a time to meet, but there was resistance and it was getting difficult. During the call, the client mentioned she was going to walk her dog, so I immediately asked if I could go with her. We had a lovely walk and talk, and at the same time I was assessing her ability to navigate roads, find her way around. Or, I have walked routes to the social/activities sessions with people, to map the area, see where it was taking the place, and then we could just pop in to check what was going on and if they would like it” (P02, social prescriber).

Together, these examples show that curiosity and creativity enable practitioners to uncover the deeper reasons behind clients’ behaviours and to engage them in flexible, meaningful ways that build trust and support.

#### Experience.

Finally, and possibly most importantly, it was reported that all the above qualities need time to be nurtured and developed among practitioners. Experience over time, including trial and error, was therefore the ultimate quality needed to support clients. As one practitioner reported: “Many skills you develop through personal experience. I’ve got little scripted pieces I use for talking with people who’ve got dementia in different situations. And those are kind of developed from my own…from my practice experience” (P03, social worker)

Because of the importance of experience, the practitioners agreed that junior practitioners should be offered support, guidance and supervision from more experienced practitioners: “As an experienced practitioner, it would be good to share and actually provide guidance, to offer opportunities for less experienced people to come and shadow you. And if you’re new to the area, to not be afraid to ask somebody who’s more experienced if they have shadowing opportunities” (P07, director of dementia home care from the private sector).

Overall, experience emerged as a vital foundation for good practice, as skills built over time—and supported through guidance and supervision from senior colleagues—enable practitioners to provide more confident, responsive, and effective care to people with dementia who live alone.

## Discussion

This study used a qualitative workshop based on co-design to collaboratively develop best-practice recommendations with practitioners supporting people with dementia who live alone without informal caregivers.

The practitioners identified a set of service- and practitioner-level recommendations to boost the quality and effectiveness of support for this population. At the service level, flexibility emerged as a fundamental requirement. Practitioners described current systems as often constrained by rigid pathways and limited options, which fail to accommodate the diverse needs of individuals without informal support. This is in line with a scoping review mapping dementia care pathways, which found that existing dementia care pathways are often highly standardised, clinically focused, and offer limited flexibility for groups whose distinct needs extend beyond traditional care practices [15].

Resistance to support in this specific client group was frequently found to reflect hidden or unspoken barriers rather than unwillingness to engage [16], highlighting the necessity of adaptable, person-centred approaches that allow practitioners to explore underlying concerns and adjust (the offer of) support accordingly. Assumptions embedded in service design—such as expectations about age, living circumstances, or functional capacity—also contributed to mismatches between available support and actual need, particularly for individuals with atypical presentations such as young-onset dementia, as also reported in previous research [17].

Alongside flexibility, time investment was identified as crucial for effective practice. Building rapport with clients who lack informal support was described as a gradual process that requires repeated, informal interactions. Everyday shared activities facilitated trust and reduced stigma more effectively than formal assessments. In line with current evidence [18], practitioners therefore emphasised the value of formally integrating relationship building into rehabilitation plans, enabling a more holistic and personable mode of engagement.

The second set of pre-requisites identified in this study are interpersonal qualities of practitioners. Personability and humanity were essential for establishing meaningful connections, particularly given the heightened risk of social isolation among this population. In line with findings from a recent systematic review on communication strategies [19], practitioners underscored the importance of demonstrating warmth, genuine interest, and attentiveness to each person’s preferred modes of interaction. Respect and humility further supported this relational foundation, enabling practitioners to recognise the individual’s autonomy and lived expertise, and to tailor decisions in ways that aligned with personal preferences and identities. The importance of retaining control and a sense of agency aligns with Kitwood’s foundational work on person-centred approaches [20], which defines personhood as being nurtured through recognition, respect, and trust, requiring practitioners to relate with clients with humility and avoid assuming practitioner superiority.

A balanced approach to sensitivity and honesty in communication was also important, ensuring transparency while minimising anxiety and preventing disengagement [19]. In addition, curiosity and creativity allowed practitioners to explore the motivations underpinning behaviours and to identify opportunities for engagement that were responsive to real-world contexts. Finally, experience was viewed as an overarching prerequisite that underpins the development of all other skills; practitioners highlighted the importance of structured support, supervision, and opportunities for shadowing to enable less experienced practitioners to cultivate these competencies over time [21].

An integrative approach to study findings suggests that practitioner-level recommendations can be put in practice consistently in routine care, only if they are enabled by organisational structures (e.g., resources). Institutional Theory [22] can be used to illustrate why practitioners who support people with dementia—particularly those living alone and without informal caregivers—may find themselves constrained despite wanting to deliver flexible, person-centred care. The theory proposes that practitioner behaviour is shaped by institutional pressures such as organisational policies, protocols, compliance requirements, and cultural expectations about “how things are done” and “what can be done”. These institutional norms and expectations strongly influence what practitioners feel permitted or able to do, thus limiting opportunities for personalised, relational, and adaptive approaches to care, even when their importance is evident. Seen through this lens, the service-level recommendations identified in this study are pre-requisites that enable practitioners to also fulfil their full professional potential.

This study was characterised by a number of strengths and limitations. While the sample was limited in its size, a diverse range of professions supporting this population was represented, including social prescribers, specialist nurses, admiral nurses, social workers, occupational therapists working in diverse range of statutory and non-statutory services including hospitals, general practice, local councils, charities and the private sector. This provided a wide spectrum of perspectives. Unfortunately, no people living with dementia were identified to take part as a research participant in the study, although, people with lived experience were involved in the study advisory group, which met regularly to steer the study and validate its findings.

This study presents important implications for service improvement. Failing to recognise the hierarchical relationship between macro-level organisational conditions and micro-level relational practice carries important implications for dementia care. When services overlook how structural enablers—such as staffing levels, supervision, continuity, and organisational culture—shape practitioners’ relational capacity, the burden of delivering person-centred, emotionally attuned care falls unfairly on individual workers. This disconnect risks eroding the relational quality that is fundamental to person-centred and relationship-centred approaches, as overstretched practitioners are left without the time, support, or reflective space required to enact warmth, sensitivity, and personalised engagement. Over time, practitioners may experience moral distress and burnout, leading to reduced workforce stability and diminished opportunities for junior staff to develop the competencies necessary for high-quality relational care. At the level of the person with dementia, insufficiently supported interpersonal practice can result in inconsistent communication, increased anxiety, loss of trust, and reduced opportunities for autonomy and agency—outcomes that run counter to the principles of personhood outlined in Kitwood’s model [20]. Ultimately, neglecting the interdependence of micro- and macro-level factors risks perpetuating task-oriented and risk-averse care cultures, undermining both practitioner wellbeing and the relational foundations essential to offer equitable care and support to those with dementia who live alone and have no one else to provide it.

## Conclusion

This study highlights that supporting people with dementia who live alone and have no informal support requires recognising the interdependence of relational practice and organisational conditions. Embedding these recommendations requires dementia services to move beyond standardised, task-focused models towards care that prioritises flexibility, continuity, and time for relationship-building. These structural adjustments enable practitioners to deliver relational, person-centred care consistently, rather than relying on individual effort alone. For people living with dementia who also live alone, these prerequisites are particularly critical because informal caregivers, who typically coordinate care, facilitate access, and interpret needs, are absent. In this context, services must assume these roles, requiring more adaptive pathways, sustained engagement, and stronger practitioner–client relationships. As a result, these conditions are not mere care enhancements, but essential foundations to achieve equitable, effective care for this especially vulnerable and underserved group of people living with dementia.
